# Post-conflict mental health needs: a cross-sectional survey of trauma, depression and associated factors in Juba, Southern Sudan

**DOI:** 10.1186/1471-244X-9-7

**Published:** 2009-03-04

**Authors:** Bayard Roberts, Eliaba Yona Damundu, Olivia Lomoro, Egbert Sondorp

**Affiliations:** 1 Conflict and Health Programme, Health Policy Unit, Department of Public Health and Policy, London School of Hygiene and Tropical Medicine, Keppel Street, London, WC1 7HT, UK; 2 Social and Demographic Statistics Department, Southern Sudan Commission for Census, Statistics and Evaluation, Juba, Southern Sudan; 3 Directorate of Research, Planning and Health Systems Development, Ministry of Health, Government of Southern Sudan, Juba, Southern Sudan

## Abstract

**Background:**

The signing of the Comprehensive Peace Agreement in January 2005 marked the end of the civil conflict in Sudan lasting over 20 years. The conflict was characterised by widespread violence and large-scale forced migration. Mental health is recognised as a key public health issue for conflict-affected populations. Studies revealed high levels of post-traumatic stress disorder (PTSD) amongst populations from Southern Sudan during the conflict. However, no studies have been conducted on mental health in post-war Southern Sudan. The objective of this study was to measure PTSD and depression in the population in the town of Juba in Southern Sudan; and to investigate the association ofdemographic, displacement, and past and recent trauma exposure variables, on the outcomes of PTSD and depression.

**Methods:**

A cross-sectional, random cluster survey with a sample of 1242 adults (aged over 18 years) was conducted in November 2007 in the town of Juba, the capital of Southern Sudan. Levels of exposure to traumatic events and PTSD were measured using the Harvard Trauma Questionnaire (original version), and levels of depression measured using the Hopkins Symptom Checklist-25. Multivariate logistic regression was used to analyse the association ofdemographic, displacement and trauma exposure variables on the outcomes of PTSD and depression. Multivariate logistic regression was also conducted to investigate which demographic and displacement variables were associated with exposure to traumatic events.

**Results:**

Over one third (36%) of respondents met symptom criteria for PTSD and half (50%) of respondents met symptom criteria for depression. The multivariate logistic regression analysis showed strong associations of gender, marital status, forced displacement, and trauma exposure with outcomes of PTSD and depression. Men, IDPs, and refugees and persons displaced more than once were all significantly more likely to have experienced eight or more traumatic events.

**Conclusion:**

This study provides evidence of high levels of mental distress in the population of Juba Town, and associated risk-factors. Comprehensive social and psychological assistance is urgently required in Juba.

## Background

The signing of the Comprehensive Peace Agreement in January 2005 marked the end of the 20 year civil conflict in Sudan between the Government of Sudan in the north and rebel movements in southern Sudan led by the Sudan People's Liberation Army/Movement. This conflict marked a continuation of the 1955–1972 war between the south and north and was rooted in long-term political, economic and cultural grievances between the south and the Government of Sudan.

Approximately 1.9 million people were killed during the 20 year conflict by violence, disease and starvation. Up to four million people were forcibly displaced from their homes as internally displaced persons (IDPs) and they went mainly to Khartoum in the north, central Sudan, or the towns of Southern Sudan. There were also up to one million refugees, living mainly in camps and cities in Kenya, Uganda, Central Africa Republic, Ethiopia, Egypt and other neighbouring countries. The majority of these displaced persons have now returned to Southern Sudan.

The challenges faced in maintaining security, fostering political stability and developing economic growth in post-conflict societies are many and complex, and are particularly acute in Southern Sudan given the longevity and severity of the war, and impoverishment of the general population and returning displaced population [1]. The ability of the government to meet the basic needs, safety and security of the population was limited. From a health perspective, Southern Sudan is marked by extremely high health needs and limited health service provision [2,3]. The health system had virtually collapsed because of the war. In 2004 it was estimated that there were between 82 and 100 doctors in Southern Sudan, equating to one doctor for every 70,000 people [4]. There remains a serious lack of health staff, facilities, equipment, supplies and medicines.

Mental health is recognised as a key public health issue for conflict-affected populations [5,6]. People experiencing poor mental health suffer substantial distress, and may be more vulnerable to violence, suicidality, and poor physical health and harmful health practices such as substance abuse. High levels of poor mental health can affect the ability of individuals, communities and societies to function both during and after conflict. Studies have also explored how exposure to traumatic events and high levels of mental distress may influence respondent attitudes to reconciliation in post-conflict societies [7,8].

Elevated rates of mental distress have been recorded amongst diverse adult populations that have experienced war. This can be either general measures of mental health,[9] or specific conditions of which the most commonly researched tend to be post-traumatic stress disorder (PTSD) and depression [10,11]. In neighbouring Uganda, reported rates of PTSD and depression amongst IDPs have varied between 75.3% and 54.3%, and 44.5% to 67.4%, respectively [8,12]. Amongst Guatemalan refugees in Mexico, rates of PTSD and depression were recorded at 11.8% and 38.8% respectively [13]. Karenni refugees living in the Thai-Burma border recorded rates of 4.6% and 41.8% of PTSD and depression [14]. A survey of Bosnian refugees in Croatia diagnosed PTSD and depression in 5.6% and 18.6% of respondents. Studies in post-conflict situations have also recorded high rates of PTSD and depression. For example, rates of PTSD in Afghanistan have varied from 20.4% to 42.1% and rates for depression from 38.5% to 67.7% [15,16]. Factors that may affect mental health outcomes include gender, exposure to traumatic events, experience of forced displacement, poverty, living conditions and access to basic goods and services [17].

A study on PTSD conducted during the conflict in southern Sudan by Karnakara, Neuner *et al *recorded PTSD rates of 48% amongst of residents in southern Sudan and 46% amongst refugees from southern Sudan living in Uganda [18]. The study also investigated factors associated with PTSD, and these included gender, age, education level, being displaced, household possessions, job type, witnessing of traumatic events, and frequency of trauma exposure [18,19].

No studies have been conducted on PTSD or other mental health outcomes in post-war Southern Sudan. The objective of this study was to measure PTSD and depression in the population in the town of Juba in Southern Sudan; and to investigate the association ofdemographic, displacement, and past and recent trauma exposure variables, on the outcomes of PTSD and depression.

## Methods

The study took place in November 2007 in Juba Town, the capital of Southern Sudan. Juba was selected because there were high numbers of IDPs, returned IDPs and refugees which facilitated investigation into the effects of forced displacement upon mental health. The estimated population of Juba at the time of the study was 150,000 people and this was increasing as refugees and IDPs returned to Juba, and other people voluntarily migrated to the town in the search for employment and better security and education and health services. As a result, there were increasing numbers of informal settlements in Juba which were characterised by poor housing water and sanitation. It was estimated that around 56% of the population in Juba were from Bari-speaking ethnic groups, 13% were Muru, and 6% were Madi [20]. Access to health services was generally better than elsewhere in Southern Sudan as Juba is served by a main district hospital and a number of smaller hospitals and health centres run by non-governmental organisations. The security situation was generally safe in Juba around the time of the study.

A cross-sectional survey design was used. The sampling population included adults aged 18 years and over living in Juba Town. The study questionnaire included the SF-8 instrument which measures general physical and mental health on a continuous measurement scale and the sample size was calculated to detect associations of independent variables on this continuous outcome scale (the findings of the SF-8 are to be presented elsewhere). The sample size required adequate power (80%) to detect conceptually important differences (0.8 standard deviation) in the health outcomes of different respondent groups within a multivariate analysis with a significance level of 5% with the size of the smallest sub-group of respondents at 5% [11]. Due to the cluster sampling method used, a design effect of 2.0 was included which doubled the required sample size [12]. The expected proportion of unusable questionnaires was set at 20%. The resultant sample size required was calculated to be a minimum of 1160.

A multi-stage cluster sampling method following previously described techniques was used as random and systematic sampling methods were not feasible due to limited data on the population of Juba town [21]. The first stage was to randomly select 36 clusters in Juba town. 36 clusters were selected, rather than the more common use of 30 clusters, to reduce the design effect [22]. The 36 clusters were selected using the probability proportional to size method. The sampling frame was a list of the 30 individual bomas (administrative districts) in Juba town with a corresponding running cumulative population size for each boma. This list was provided by the Southern Sudan Commission for Census, Statistics and Evaluation from their preliminary census estimates of October 2006. This was considered to be the most accurate population data available for Juba Town. The clusters were then allocated to bomas proportionally to their population sizes following the probability proportional to size technique to ensure self-weighting [21]. The 36 clusters were allocated to 23 of the 30 bomas in Juba town using this process. The total population living in the 23 bomas was estimated as 131,194 of which 64,416 were estimated to be of eligible age (≥18 years).

The second stage consisted of randomly choosing individuals from the selected clusters. As the clusters were already self-weighted, the same number of individuals were chosen from within each of the selected clusters. The Expanded Programme on Immunisation (EPI) random walk method was used to help randomly select households for this stage. This involved going to the centre of each boma and then following an imaginary line drawn from the centre to the edge of the boma in a direction determined by spinning a pen. Households along this imaginary line were then counted and a household randomly selected using a random number list. Subsequent households were then selected which were two structures removed from the previously selected structure based on a principal of proximity [22,23].

A questionnaire was developed consisting of items on the demographic, displacement and socio-economic characteristics of the respondents, and existing health measurement instruments to measure trauma exposure, PTSD and depression (and general health status using the SF-8 for which the results are to be presented elsewhere). An adapted version of the original Harvard Trauma Questionnaire (HTQ) was used to identify exposure to trauma events [24]. This consisted of 16 questions with a 'yes/no' response on exposure to traumatic event types throughout the respondent's lifetime and also within the previous 12 months. PTSD was measured using 30 questions on trauma symptoms with a 4 point severity scale and a recall period of 1 week. The first 16 items are based upon the *Diagnostic and Statistical Manual for Mental Disorders, Fourth Edition *(DSM-IV), and the remaining 14 items were developed specifically for forcibly displaced and conflict-affected populations [24,25]. Mean PTSD scores ≥ 2.0 were considered significant for meeting symptom criteria of PTSD based upon the instrument standards [26]. Scores for symptoms of depression were measured using the 15 depression items from the Hopkins Symptoms Check List-25 (HSCL-25) [26,27]. This also has a four point severity scale and a recall period of one week. Mean depression scores ≥ 1.75 were considered significant for meeting symptom criteria of depression based upon the instrument standards [26]. The 15 depression items are consistent with the depression items in the DSM-IV [25,27]. The validity of the HTQ and HSCL-25 have been tested and proven for use with displaced persons in a number of countries [8,13,24,27,28].

The questionnaire was translated and delivered in Juba Arabic and Bari, the two main language in Juba town. The translation followed recommended guidelines was conducted by experienced translators [24,26]. A review of the forward and back translations and pre-testing was conducted by the study staff to ensure that the meanings and concepts of the questionnaire items remained consistent with the original English version.

20 data collectors were recruited for the survey (11 women and 9 men). Their average age was 29 years. They all spoke fluent Juba Arabic and/or Bari and all had experience of survey data collection in Juba. One week's intensive training was provided for the study and included: the background, aims and intended value of the study; mental health and research, focusing on PTSD and depression; detailed review of the individual items in the survey; ethical issues of informed consent, confidentiality, and anonymity; and ensuring high interview quality and avoiding bias. The data collectors did not have clinical knowledge related to PTSD and depression but the HTQ and HSCL-25 are designed to be used by lay interviewer who have received training [26]. Supervision and quality control were provided by four team leaders and two members of the study staff.

The data collection took place between 20 and 30 November 2007. The interviews took an average of 42 minutes to complete the questionnaire items. A signed consent form was used to ensure informed consent and clarify that no direct benefit could be expected from participating in the study. All data collected was confidential and anonymous. Ethical approval for the study was provided by the Ministry of Health of the Government of Southern Sudan, and the London School of Hygiene and Tropical Medicine.

Two data entry clerks were used to enter the data into EpiData Entry version 3.2 (DK-5230, Odense, Denmark). Questionnaires were cross-checked by study staff and analysis conducted of the dataset to check for inconsistent data entries. All analysis was performed using STATA version 10 (Stata Corporation, College Park, Texas, USA) and adjusted for the clustered design. The outcome of PTSD was dichotomised into respondents exhibiting or not exhibiting signs of PTSD (cut off ≥ 2.0), and the outcome of depression dichotomised into respondents exhibiting not exhibiting signs of depression (cut off ≥ 1.75), based upon the cut off levels given in the instrument guidelines [26]. The Cronbach alpha for internal consistency reliability was tested and estimated at 0.92 for the PTSD scale and 0.85 for the depression scale, above the suggested minimum threshold level for internal reliability coefficient of ≥ 0.70 [29]. Multivariate logistic regression was applied to produce odds ratios (OR) of associations between independent demographic, displacement and trauma exposure variables with outcomes of PTSD and depression and adjusted to address the influence of the other significant independent variables. Initially, all the independent variables were included in a univariate analysis to explore associations between each individual independent variables and outcomes of PTSD and depression. The variables which had a statistically significant (*P *< 0.05) association with these outcomes in the univariate analysis were then included for the multivariate analysis which followed a backward elimination regression approach. Only the associations in the multivariate analysis which were statistically significant (*P *< 0.05) were included in the final results. Interaction tests were conducted to explore if the effect of one independent variable significantly varied (P < 0.05) according to the level of the effects of another independent variable within the overall regression model. However, no significant interaction effects were found. The same process for multivariate analysis was also applied to look for demographic and displacement variables associated with exposure to traumatic events.

## Results

1242 interviews were completed and the overall response rate was 96.2%. Table 1 presents the sampling profile of the survey.

**Table 1:** Sampling profile

	**Juba Town**
Bomas visited	23
Absent individuals	32
Non-consenting individuals	13
Incomplete individual interviews	4
Completed individual interview	1242

### Sample characteristics

The sample characteristics are provided in Table 2. Women and men were equally represented in the sample. The mean age of respondents was 33 years. 49.2% of respondents were from Bari-speaking ethnic groups, 11.9% were Muru, and 6.0% were Madi. 35.9% of respondents had either previously been forcefully displaced or were still forcefully displaced from their home areas. 12.6% of respondents had experienced forced displacement outside Sudan as refugees. 10.0% of respondents had previously experienced forced displacement within Sudan as IDPs. 12.6% of respondents were still displaced as IDPs. The principal reason cited for preventing their return home was insecurity (21.3%), followed by fear of landmines in home areas (19.2%), and lack of jobs and income in their home areas (17.8%). 12.3% of respondents had been displaced more than once.

**Table 2:** Sample characteristics of Juba health survey respondents (N = 1242)

**Characteristics**	**Number (%)**
Number of women	630 (50.7)

Age, mean	33 years

**Religion**	
Christian	1142 (91.9)
Muslim	93 (7.5)
other	7 (0.6)

**Ethnicity**	
Bari Speaking	611(49.2)
Muru	148(11.9)
Madi	74 (6.0)
other	409(32.9)

**Marital status**	
married	912 (73.4)
single	249 (20.0)
divorced/separated	19 (1.5)
widowed	57 (4.6)
forcefully separated	5 (0.4)

**Education level**	
never attended school	331(26.7)
completed or partially primary school	393 (31.6)
completed or partially completed secondary school	380 (30.6)
Completed post-secondary school (eg. college)	138 (11.1)

**Displacement Characteristics**	
never forcibly displaced	797 (64.2)
previously displaced as refugee	156 (12.6)
previously displaced as IDP	124 (10.0)
currently IDP	156 (12.6)
previously both refugee and IDP	9 (0.7)
displaced more than once	253 (12.3)

Abbreviations: IDP, internally displaced person

### Exposure to Trauma

The results on exposure to traumatic events are given in Table 3. 92.4% of respondents had ever experienced 1 or more of the 16 trauma events covered in the questionnaire. 22.7% of respondents had ever experienced 8 or more of the 16 trauma events covered in the questionnaire. 51.5% of the respondents had ever directly experienced a combat situation. 49.6% had witnessed the murder of family or friends. 20.1% of respondents reported having been beaten or tortured. Social deprivation is evidenced by the fact that 64.7% of respondents had ever lacked food or water, and 47.9% had been seriously ill without access to medical care. The most common trauma events experienced within the previous 12 months from the survey date were lacking food or water (29.2%); being seriously ill without access to medical care (24.5%), and lack of housing or shelter (13.0%).

**Table 3:** Exposure to traumatic events, by timeframe (N = 1242)

**Trauma event**	**Number of persons experiencing event**		
	Event <12 months of survey N (%)	Event ≥ 12 months of survey N (%)	Total N (%)
Lack of food or water	363 (29.23)	440 (35.43)	803 (64.65)
Unnatural death of family/friend	184 (14.81)	558 (44.93)	742 (59.74)
Combat situation	89 (7.17)	550 (44.28)	639 (51.45)
Murder of family/friend	141 (11.35)	475 (38.24)	616 (49.60)
Very ill without medical care	304 (24.48)	291 (23.43)	595 (47.91)
Lack of housing or shelter	162 (13.04)	325 (26.17)	487 (39.21)
Being close to death	97 (7.81)	338 (27.21)	435 (35.02)
Forced separation from family	70 (5.72)	250 (20.13)	320 (25.76)
Serious injury	109 (8.78)	210 (16.91)	319 (25.68)
Tortured or beaten	70 (5.64)	180 (14.49)	250 (20.13)
Forced isolation from others	73 (5.88)	160 (12.88)	233 (18.76)
Murder of stranger or strangers	50 (4.03)	181 (14.57)	231 (18.60)
Forced to accept thoughts against will	52 (4.19)	119 (9.58)	171 (13.77)
Imprisonment	43 (3.46)	125 (10.06)	168 (13.53)
Being abducted or kidnapped	17 (1.37)	85 (6.84)	102 (8.21)
Rape or sexual abuse	21 (1.69)	61 (4.91)	82 (6.60)

Men generally reported higher rates of exposure to individual trauma events than women (Table 4). Men also reported higher exposure to 8 or more of the 16 trauma events included in the questionnaire (27.8% [95% CI 23.0–33.1] of men, compared to 17.8% [95% CI 14.0–22.3] of women).

**Table 4:** Exposure to traumatic events, by gender (N = 1242)

**Trauma event**	**Men (N = 612)**		**Women (N = 630)**		**Total (N = 1242)**
	N (%)	[95% CI]	N (%)	[95% CI]	N (%)
Lack of food or water	408 (66.67)	[61.12–71.79]	395 (62.70)	[56.51–68.49]	803 (64.65)
Unnatural death of family/friend	387 (63.24)	[56.84–69.20]	355 (56.35)	[48.21–64.16]	742 (59.74)
Combat situation	337 (55.07)	[48.78–61.19]	302 (47.94)	[42.50–53.42]	639 (51.45)
Murder of family/friend	336 (54.90)	[48.31–61.33]	280 (44.44)	[38.20–50.87]	616 (49.60)
Very ill without medical care	291 (47.55)	[42.37–52.79]	304 (48.25)	[43.96–52.57]	595 (47.91)
Lack of housing or shelter	258 (42.16)	[35.94–48.63]	229 (36.35)	[31.28–41.75]	487 (39.21)
Being close to death	230 (37.58)	[32.37–43.09]	205 (32.54)	[27.19–38.39]	435 (35.02)
Forced separation from family	181 (29.58)	[26.10–33.31]	139 (22.06)	[17.43–27.51]	320 (25.76)
Serious injury *	193 (31.54)	[26.53–37.01]	126 (20.00)	[16.64–23.85]	319 (25.68)
Tortured or beaten *	157 (25.65)	[21.54–30.25]	93 (14.76)	[11.78–18.34]	250 (20.13)
Forced isolation from others	135 (22.06)	[18.31–26.33]	98 (15.56)	[11.96–19.98]	233 (18.76)
Murder of stranger or strangers *	136 (22.22)	[19.18–25.60]	95 (15.08)	[11.83–19.04]	231 (18.60)
Forced to accept thoughts against will	92 (15.03)	[12.00–18.67]	79 (12.54)	[9.96–15.67]	171 (13.77)
Imprisonment *	107 (17.48)	[13.93–21.71]	61 (9.68)	[7.39–12.60]	168 (13.53)
Being abducted or kidnapped	59 (9.64)	[7.54–12.25]	43 (6.83)	[5.26–8.81]	102 (8.21)
Rape or sexual abuse	31 (5.07)	[3.59–7.10]	51 (8.10)	[5.85–11.11]	82 (6.60)

* Statistically significant (*P *< 0.05) difference between men and women

### Prevalence of PTSD and Depression

36.2% of respondents met symptom criteria for PTSD (Table 5). The PTSD rates were 42.5% amongst women and 29.7% amongst men. 49.9% of respondents met symptoms for depression (Table 5). The depression rates were 58.7% amongst women and 40.9% amongst men.

**Table 5:** Prevalence rate of symptoms of PTSD and depression (N = 1242)

	**PTSD**		**Depression**	
	N (%)	[95% CI]	N (%)	[95% CI]
**Total**	450 (36.23)	[33.16–39.42]	620 (49.92)	[46.53–53.31]
women	268 (42.54)	[39.42–45.72]	370 (58.73)	[55.19–62.18]
men	182 (29.74)	[25.35–34.54]	250 (40.85)	[36.69–45.14]

Abbreviations: PTSD, post-traumatic stress disorder; CI, confidence interval.

Symptoms of PTSD (HTQ PTSD Score = ≥2.0) and depression (HSCL score = ≥1.75).

### Multivariate Analysis

The statistically significant (*P *< 0.05) results of the multivariate logistic regression analysis on the association of key demographic, displacement and trauma exposure variables on the outcomes of PTSD (cut off ≥ 2.0) and depression (cut off ≥ 1.75) are presented in adjusted odds ratios in Table 6. These show a strong association of gender on mental distress, with women over twice as likely as men to exhibit symptoms of PTSD (OR 2.01 [95% CI 1.52–2.66]) and depression (OR 2.37 [95% CI 1.91–2.94]). Respondents who were no longer married (divorced/separated, widowed, or forcefully separated) were more than twice as likely to exhibit symptoms of PTSD (OR 2.10 [95% CI 1.28–3.44]) than respondents who were married or had never been married. Being forcefully displaced two or more times (compared to once or never displaced) was associated with PTSD (OR 1.81 [95% CI 1.18–2.76]) and depression (OR 2.22 [95% CI 1.70–2.89].

**Table 6:** Multivariate analysis of variables associated with symptoms of PTSD and depression

**Variable**	**PTSD (N = 450)**		**Depression (N = 620)**	
	OR [95% CI]	*P*	OR [95% CI]	*P*
Sex (women compared to men)	2.01 [1.52–2.66]	<0.01	2.37 [1.91–2.94]	<0.01
No longer married∞	2.10 [1.28–3.44]	0.01		
Forcefully displaced more than once†	1.81 [1.18–2.76]	0.01	2.12 [1.45–3.09]	<0.01
Forcefully separated from family	1.91 [1.38–2.64]	<0.01	1.80 [1.28–2.54]	<0.01
Experiencing very ill health without medical care	1.89 [1.41–2.53]	<0.01	2.22 [1.70–2.89]	<0.01
Being injured	1.87 [1.49–2.36]	<0.01	1.50 [1.15–1.94]	<0.01
Experienced ≥8 trauma events in lifetime ‡	2.44 [1.76–3.37]	<0.01	2.21 [1.60–3.07]	<0.01
Experienced ≥4 trauma events within 12 months‡	2.89 [1.89–4.41]	<0.01	2.09 [1.32–3.32]	<0.01

Abbreviations: CI, confidence interval; PTSD, post-traumatic stress disorder; OR, odds ratio (adjusted).

Symptoms of PTSD (HTQ PTSD Score = ≥2.0) and depression (HSCL score = ≥1.75).

Statistically significant (*P *< 0.05) demographic, displacement and all individual trauma exposure variables (Table 3) from univariate analysis included in multivariate analysis. Only significant (*P *< 0.05) adjusted odds ratios from multivariate analysis presented.

∞ No longer married (divorced/separated, widowed, forcefully separated) compared to married and never married;

† Displaced more than once (refugees and IDPs) compared to displaced once or never displaced.

‡ Frequency of trauma events analysed separately from individual trauma exposure variables in multivariate analysis.

The individual trauma events with significant associations with PTSD were being forcefully separated from family (OR 1.91 [95% CI 1.38–2.64]), experiencing very ill health without access to medical care (OR 1.89 [95% CI 1.41–2.53]), and being injured (OR 1.87 [95% CI 1.49–2.36]). It was the same trauma exposure events which showed a significant association with depression: being forcefully separated from family (OR 1.80 [95% CI 1.28–2.54]); experiencing very ill health without access to medical care (OR 2.22 [95% CI 1.70–2.89]); and being injured (OR 1.50 [95% CI 1.15–1.94]). Respondents who had experienced 8 or more of the 16 trauma events included in the questionnaire were more likely to exhibit symptoms of PTSD (OR 2.44 [95% CI 1.76–3.37]) and depression (OR 2.21 [95% CI 1.60–3.07]). Respondents who had experienced four or more of the 16 trauma events within 12 months of the survey date were also more likely to exhibit symptoms of PTSD (OR 2.89 [95% 1.89–4.41]) and depression (OR 2.09 [95% 1.32–3.32]).

A multivariate analysis was also conducted to investigate which sub-groups within the sample were more vulnerable to experiencing eight or more traumatic events. Men, IDPs, and refugees and persons displaced more than once were all significantly more likely to have experienced eight or more traumatic events (Table 7).

**Table 7:** Multivariate analysis of variables associated with trauma exposure (N = 282)

**Variable**	**Exposure to ≥8 trauma events**	
	OR [95% CI]	*P*
Sex (men compared to women)	1.66 [1.27–2.17]	
IDP ∞	2.71 [1.64–4.46]	<0.01
Refugee ±	2.89 [1.77–4.73]	<0.01
Forcefully displaced more than once†	2.03 [1.23–3.35]	<0.01

Abbreviations: CI, confidence interval; IDP, internally displaced person; OR, odds ratio (adjusted).

Statistically significant demographic and displacement variables (*P *< 0.05) from univariate analysis included in multivariate analysis. Only significant (*P *< 0.05) adjusted odds ratios from multivariate analysis presented.

∞ IDPs (past and present combined) compared to all non-IDPs.

± Refugees compared to all non-refugees.

† Displaced more than once (refugees and IDPs) compared to displaced once or never displaced.

## Discussion

This is the first study to be conducted on mental health in post-conflict Southern Sudan, and contributes to evidence on mental health from southern Sudan from during the conflict [18,19]. Over one third (36.2%) of respondents met symptom criteria for PTSD and half (49.9%) of respondents met symptom criteria for depression. These compare with PTSD rates of 48% amongst of residents in southern Sudan and 46% amongst refugees from southern Sudan living in Uganda recorded in the study by Karunakara *et al *[18]. Rates of depression have not been previously researched in Southern Sudan.

The study revealed high exposure to trauma among the population of Juba, with nearly one quarter (22.7%) of respondents ever experiencing 8 or more of the 16 trauma events covered in the questionnaire. Importantly, the study provides evidence of the continued exposure to traumatic events amongst populations in Juba. Within the previous 12 months of the survey date, almost one third (29.2%) of respondents reported lacking food or water and one quarter (24.5%) reporting having been very ill without access to medical care. The study also provided new data on factors associated with exposure to traumatic events; with men, IDPs, refugees and persons displaced more than once all significantly more likely to have experienced eight or more traumatic events.

A number of significant associations of independent variables were found with outcomes of PTSD and depression after adjusting for effects of other demographic and trauma exposure variables. Women are at particularly high risk of poor mental health, and also people who are no longer married, as recorded in other studies on mental health of displaced populations [7,8,13,15-18,30,31]. The study by Karunakara *et al *also shows a strong association between gender and PTSD [18]. Although men reported higher exposure to traumatic events than women, men reported lower levels of mental distress. This could be due to the psychological consequences of rape, the violent loss of partner and children, and of becoming a single parent or widow, but further investigation is required to explain gender-differences in the response to traumatic events [31].

Forced displacement was associated with exposure to traumatic events, and repeated displacement was associated with both PTSD and depression. The study by Karunakara *et al *also recorded that Sudanese refugees suffered higher exposure to traumatic events and PTSD than Ugandans and Southern Sudanese who were not refugees. However, the study by Karunakara *et al *indicated that a history of forced migration reduced the risk of PTSD after controlling for age, socio-economic factors and traumatic experiences [18]. Further investigation on the specific effects of forced migration in Southern Sudan and elsewhere would be of value.

The study explored the association of individual trauma exposure variables with PTSD and depression. Evidence on the role of these individual trauma variables has not been previously documented in Southern Sudan. These variables included being forcefully separated from family, being injured, and experiencing very ill health without access to medical care. The latter variable highlights the negative effect that deprivation of basic goods and services in post-conflict Juba has upon mental health. Respondents who had experienced a high frequency of traumatic events, particularly within one year of the survey date, were also more vulnerable to PTSD and depression. Evidence on this dose-effect relationship between trauma and PTSD and depression supports the findings of other studies, including by Karunakara *et al *in Southern Sudan [11,18,19,32]. A study on IDP children in Southern Sudan also noted the association of exposure to traumatic events with poor mental health outcomes [33].

Southern Sudan continues to struggle with overwhelming health needs, poor infrastructure, limited capacity to provide essential health services, and probable funding gaps for the provision of health services[2,3,34] Currently, there are almost no mental health services in southern Sudan with one or two localised exceptions such as the activities provided by non-governmental organisations. The principal delivery mechanism of health services in Southern Sudan is through a 'basic package of health services' funded by the Government of Southern Sudan and international donors and provided by non-governmental organisations [35]. These health services need to be urgently scaled up, and mental health activities incorporated into services provided through the basic package of health services. These activities could include training of health care staff, and community workers to provide basic support and referral services, and to organise community-based self-help support groups [36]. Improved security and the provision of other social and economic services are also urgently required to reduce exposure to factors which may increase poor mental health outcomes.

Without improved services and protection, it is likely that there will continue to be high levels of poor mental health causing significant distress, and associated poor physical health and harmful health practices. The social functioning of individuals and ability to support themselves and their families may also be impacted. Studies from other countries have also explored how exposure to traumatic events and high levels of mental distress may influence respondent attitudes to reconciliation in post-conflict societies [7,8]. While this issue may not be generally too relevant in Southern Sudan where the majority of the ex-belligerents live great distances away from one another, it may be pertinent in areas where there were more localised clashes, some of which continue to take place. Further research is required on this issue

### Limitations

This study has a number of limitations. Firstly, concerns exist over the use of metrics for analysing specific mental health conditions of war-affected populations in different cultural settings, including that they may over-estimate symptom scores, particularly for PTSD, and that there may be mixing of anxiety and depression symptoms which could result in over-estimated depression scores [37-41]. The questionnaire items also took on average of 42 minutes to administer per respondent and this short interview duration could have potentially impacted upon the validity of the health measurement instruments, particularly as interviews were carried out by trained lay-interviewers. The HTQ and HSCL-25 used in this study have been specifically developed and validated for conflict-affected populations and have been used widely in a diverse range of cultural settings [8,13,15,24,27,28]. The instruments also had high internal reliability levels in this study using the Cronbach alpha test. However, further research is required to establish the sensitivity and specificity of mental health diagnostic instruments in Southern Sudan and other conflict-affected areas [24,39,42,43].

Secondly, the study was unable to consistently match the gender of interviewer and respondents. As a result, there may have been underreporting of certain sensitive traumatic events such as the item of rape or sexual violence. In this study, 6.6% of respondents reported having ever experienced rape or sexual violence, with 1.7% having experienced it in the previous one year. These rates compare with the study by Karunakara *et al *in which 2.4% of Sudanese nationals and 9.8% for Sudanese refugees reported having ever experienced rape; and 0.2% of Sudanese nationals and 5.1% of Sudanese refugees in the past year [18]. An HIV Behavioural Surveillance Survey conducted in Juba in late 2006 reported that 3% of women and no men said that they had ever been forced to have sex [20]. This study also did not explore different types of sexual violence such as forced marriage or forced prostitution through separate questionnaire items and so the study results may also not adequately reflect the different types of sexual violence experienced by respondents.

Thirdly, this study did not explore the relationship between PTSD and depression with coping, functioning and social relations. Further research is required is required to investigate how mental distress may affect coping and social functioning in Southern Sudan.

Fourthly, there is a high degree of mobility in the population of Juba town which would have reduced the accuracy of the sampling frame and the sampling process. However, the sampling frame source was felt to be the most accurate data source available. Lastly, the cross-sectional design means only associations can be described between variables rather than attributing causation.

## Conclusion

This study provides important evidence on the prevalence of mental distress and associated risk factors in Juba in Southern Sudan. Further research is required to explore the findings and appropriate responses. Some evidence exists on the effectiveness of mental health interventions in resource poor and conflict affected settings [6,44-46]. However, limited resources are provided for mental health services in low-income countries, and the needs are particularly acute in countries emerging from conflict such as Southern Sudan [47,48]. Similarly, greater resources are required for broader support services such as health services, food and water, housing and security in post-conflict countries. Without such resources, the individual and social well-being and functioning in post-war Southern Sudan may be seriously compromised.

## Abbreviations

(CI): Confidence Interval; (HTQ): Harvard Trauma Questionnaire; (HSCL-25): Hopkins Symptoms Check List-25; (IDP): Internally Displaced Person; (OR): Odds Ratio; (PTSD): Post-traumatic stress disorder.

## Competing interests

The authors declare that they have no competing interests.

## Authors' contributions

BR led the study concept and design, data collection, data analysis, and drafting of the manuscript. EYD participated in study development, data collection, and review of the manuscript. OLD participated in developing the study development and design, and review of the manuscript. ES participated in developing the study concept and design, and review of the manuscript. All authors read and approved the final manuscript.

## Pre-publication history

The pre-publication history for this paper can be accessed here:


